# Induction of Antigen-Specific Tolerance in T Cell Mediated Diseases

**DOI:** 10.3389/fimmu.2020.02194

**Published:** 2020-09-29

**Authors:** Laura Passerini, Silvia Gregori

**Affiliations:** Mechanisms of Peripheral Tolerance Unit, San Raffaele Telethon Institute for Gene Therapy (SR-Tiget), IRCCS San Raffaele Scientific Institute, Milan, Italy

**Keywords:** tolerance, dendritic cells, autoimmunity, cell therapy, immunomodulation, antigen-specific

## Abstract

The development of novel approaches to control unwanted immune responses represents an ambitious goal in the management of a number of clinical conditions, including autoimmunity, autoinflammatory diseases, allergies and replacement therapies, in which the T cell response to self or non-harmful antigens threatens the physiological function of tissues and organs. Current treatments for these conditions rely on the use of non-specific immunosuppressive agents and supportive therapies, which may efficiently dampen inflammation and compensate for organ dysfunction, but they require lifelong treatments not devoid of side effects. These limitations induced researchers to undertake the development of definitive and specific solutions to these disorders: the underlying principle of the novel approaches relies on the idea that empowering the tolerogenic arm of the immune system would restore the immune homeostasis and control the disease. Researchers effort resulted in the development of cell-free strategies, including gene vaccination, protein-based approaches and nanoparticles, and an increasing number of clinical trials tested the ability of adoptive transfer of regulatory cells, including T and myeloid cells. Here we will provide an overview of the most promising approaches currently under development, and we will discuss their potential advantages and limitations. The field is teaching us that the success of these strategies depends primarily on our ability to dampen antigen-specific responses without impairing protective immunity, and to manipulate directly or indirectly the immunomodulatory properties of antigen presenting cells, the ultimate *in vivo* mediators of tolerance.

## Introduction

The identification of novel approaches designed to selectively control antigen(Ag)-specific effector T (Teff) cell responses and promote or restore tolerance in T cell mediated diseases is an unsolved issue in the management of autoimmune diseases in humans. On this line, a new version of vaccination, also called “inverse vaccination,” aims at inducing or restoring an immunological state of unresponsiveness, either toward foreign Ags (e.g., protein therapeutics, allergens, or transgenes) or autoAgs (1). The overall goal of inverse vaccination strategies is to dampen the adverse response, through deletion, inhibition or deviation of Ag-specific Teff cells, and to support the induction and/or expansion of Ag-specific T regulatory cells (Tregs). Tregs are recognized as a cell population responsible for induction and maintenance of immune tolerance. The best characterized subsets are the Forkhead box P3 expressing Tregs (FOXP3^+^ Tregs) (2) and the IL-10-producing type 1 regulatory (Tr1) cells (3).

**Figure 1:**
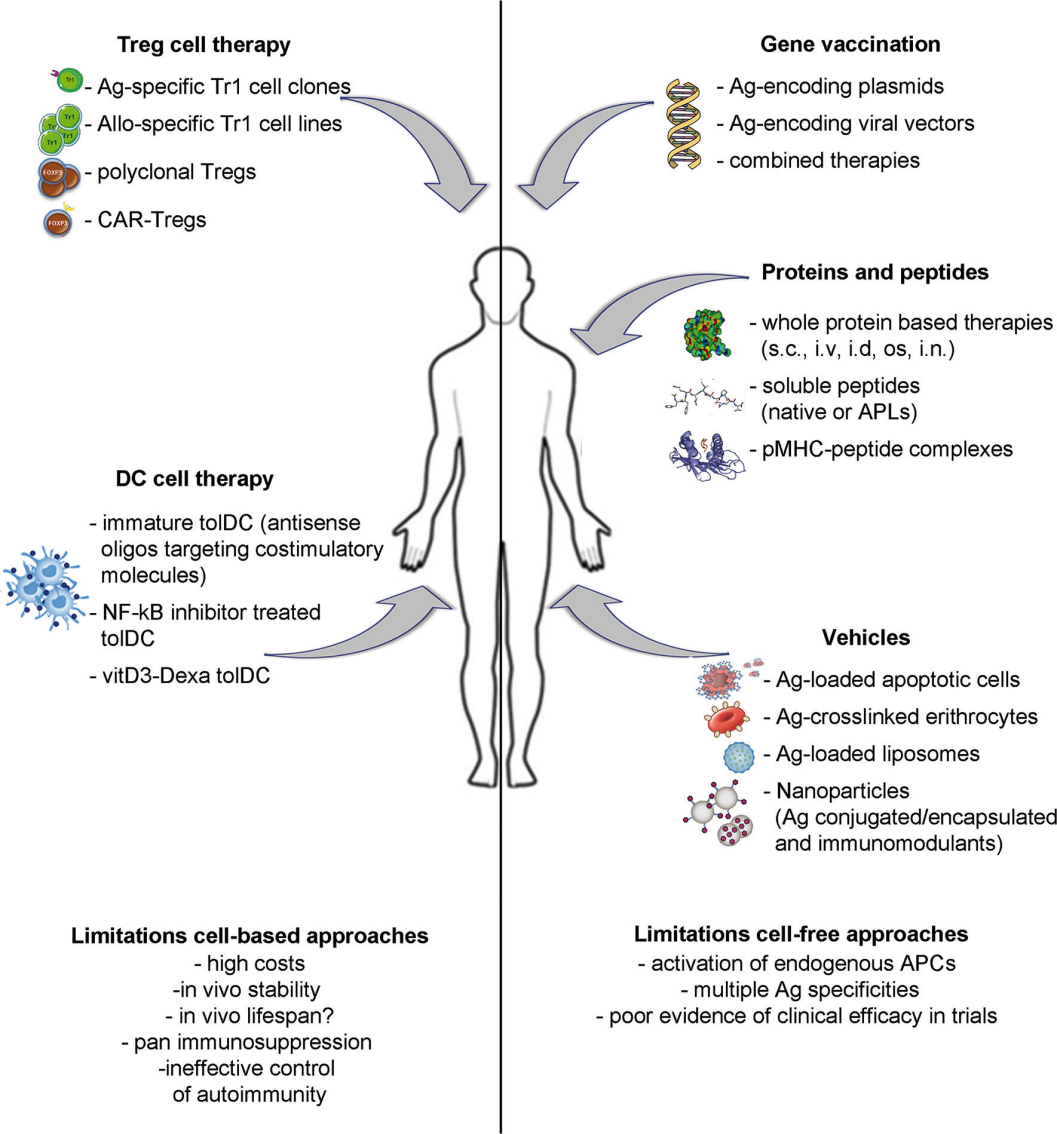
Strategies to induce Ag-specific tolerance in T cell mediated diseases. Approaches under development include: inverse vaccination with autoantigen-encoding DNA or viral vectors; *in vivo* administration of whole Ags, unmodified peptides or altered peptide ligads (APLs); autoantigen-loaded vehicles; transfer of polyclonal or Ag-specific Tregs or of tolerogenic DC loaded with disease-relevant Ags.

A number of different strategies have been proposed as inverse vaccination: (i) cell-free based approaches, including gene vaccination and protein or peptide delivery; (ii) vehicle approaches, to deliver Ags by means of apoptotic cells, liposomes, or nanoparticles; (iii) cell-based approaches, aimed at providing specialized cells to reinforce the regulatory arm of the immune system. This Review aims to provide an overview of the most promising approaches currently under development and clinical testing (Figure 1 and Table 1) and their potential advantages and limitations.

**Table 1:** Clinical trials using antigen-specific approaches.

	**Design**	**Disease**	**outcome**	**Trial ID**	**References**
Plasmid DNA	MBP, i.m.	MS Adult	Reduced IFNg-producing CD4^+^ T cells Decrease of autoantibodies in CNS	NCT00103974	(4)
	MBP, i.m.	MS Adult	No effects	NCT00382629	(5)
	hINS, i.m.	T1D Adult	Increased C-peptide Decreased insulin-reactive CD8^+^ T cells	NCT00453375	(6)
	hINS, i.m.	T1D Children	Ongoing	NCT03794960	N.A.
	hINS, i.m.	T1D Adult	Ongoing	NCT03794973	N.A.
	PPI + TGF-β1, IL-10, IL-2, s.c.	T1D Adult	Ongoing	NCT04279613	N.A.
Proteins	Ins, oral	T1D Adult	No clinical effects	N.A.	(7)
	Ins, oral	T1D Adult	No clinical effects	IMDIAB trial	(8)
	Ins, oral	T1D Adult	Increased C-peptide in patients > age 20 years	N.A.	(9)
	Ins, oral	FDR	No delay or no T1D prevention	NCT00004984	(10)
	Ins, intranasal	T1D Adult	No T1D prevention Evidence of insulin-specific tolerance	N.A.	(11)
	Ins, intranasal	FDR children	No T1D prevention	NCT00223613	(12)
	Ins, intranasal	FDR	Ongoing	NCT00336674	N.A.
	Ins, oral	FDR	No T1D prevention Modulation of insulin-response	ISRCTN76104595 isrctn.org	(13)
	Ins, oral	FDR	Ongoing	NCT02580877	N.A.
	Ins+ IFA i.m.	T1D Adult	No T1D prevention Induction of insulin-specific Tregs	NCT00057499	(14)
	Ins + MAS-1 i.m.	T1D Adult	ongoing	NCT03624062	N.A.
	GAD-alum s.c.	Newly diagnosed T1D	No clinical effects	NCT00529399	(15)
	GAD-alum s.c.	Newly diagnosed T1D	No clinical effects	NCT00723411	(16)
	GAD-alum s.c.	LADA	No clinical effects	N.A.	(17, 18)
	GAD-alum + Vit D s.c.	LADA	Ongoing	NCT04262479	N.A.
	Myelin, oral	RR-MS	No clinical effects	N.A.	(19)
	Myelin, oral	RR-MS	No clinical effects Induction of myelin-specific TGF-b1+ cells	N.A.	(20)
Peptides	PPI (C19-A3), intradermal	Newly diagnosed T1D	Maintenance of C-peptide over 6-months Increased IL-10-expressing T cells	NCT01536431	(21, 22)
	MBP8298, i.v.	Secondary progressive MS	No stable clinical benefit	NCT00468611	(23, 24)
	Multiple Islet Peptides, intradermal	Newly diagnosed T1D	Not published	NCT02620332	N.A.
	IMCY-0098, s.c.	Newly diagnosed T1D	Not published	NCT03272269	N.A.
	MBP-derived peptide cocktail ATX-MS-1467, intradermal – s.c.	RR-MS	Safety and tolerability No clinical response	NCT01097668	(25)
	MBP-derived peptide cocktail ATX-MS-1467, intradermal	RR-MS	Reduction in MRI lesions	NCT01973491	(26)
	MBP85-99, MOG35-55, and PLP139-155, transdermal	RR-MS	Reduction of clinical outcomes, induction of Tregs	N.A.	(27, 28)
	HLA-DQ2.5-restricted gliadin peptides, intradermal	CD-GFD, HLA-DQ2·5	Unresponsiveness of T cells after gluten challenge	NCT02528799	(29, 30)
	gliadin peptides, intradermal	CD-GFD, HLA-DQ2·5	Ongoing	NCT03644069	N.A.
	APL (NBI-5788), s.c.	MS	Persistent Th2 immune deviation Hypersensitivity	N.A.	(31, 32)
	APL (CGP77116), s.c.	MS	Th1 skewing Disease exacerbation in some patients	NCT00001781	(33)
	APL (NBI-6024), s.c.	Newly diagnosed T1D	No clinical effects	NCT00873561	(34)
	DR2:MBP84-102 (AG284), i.v.	Progressive MS	No clinical effects	N.A.	(35)
	DR2:MOG35,55 (RTL100), i.v.	MS Adult	No clinical effects	N.A.	(36)
Peptide coupled with cells	PBMC coupled with a pool of myelin peptides, i.v.	RR-MS Progressive MS	Decrease in antigen-specific T cells	NCT01414634 ETIMS	(37)
	RBC coupled with a pool of myelin peptides, i.v.	RR-MS	Decrease in myelin-specific T cells with an increased Treg frequency	ETIMS^Red^	(38)
Liposomes Nanoparticles	PLGA-gliadin (TIMP-GLIA) i.v.	CD	Completed results unpublished	NCT03486990	N.A.
	PLGA-gliadin (TIMP-GLIA) i.v.	CD-GFD	Ongoing	NCT03738475	N.A.
Treg-based therapy	Expanded Treg, i.v.	Newly diagnosed T1D children	Short term preservation of C-peptide No long-term effects	ISRCTN06128462 isrctn.org	(39, 40)
	Expanded Treg, i.v.	Newly diagnosed T1D	Short term preservation of C-peptide	NCT01210664	(41)
	Expanded Treg, i.v.	SLE	Terminated due to participant recruitment	NCT02428309	(42)
	Expanded Treg, i.v.	Newly diagnosed T1D	Completed, unpublished	NCT02691247	(42)
	Expanded Treg, i.v.	Autoimmune Hepatitis	Ongoing	NCT02704338	(42)
	Expanded Treg, i.v.	Pemphigus Vulgaris	Ongoing	NCT03239470	(42)
	Expanded Treg, i.v.	IBD	Ongoing	NCT03185000	(42)
	Expanded Treg, i.v.	Alzheimer Disease	Ongoing	NCT03865017	N.A.
	Treg, intravitreous	Bilateral Severe Uveitis	Suspended	NCT02494492	(42)
	Cord-blood Treg, i.v.	Guillain–Barré syndrome	Ongoing	NCT03773328	N.A.
	Expanded Treg + IL-2, i.v.	Newly diagnosed T1D	Ongoing	NCT02772679	N.A.
	Expanded Treg + Liraglutide, i.v.	Newly diagnosed T1D	Ongoing	NCT03011021	N.A.
	Ova-specific Tr1 cell clones, i.v.	Refractory Crohn's	Expansion of OVA-specific Treg Limited clinical responses	CATS1/CATS29	(43)
DC-based therapy	shRNA CD40, CD80 and CD86, i.p.	T1D	Safety and tolerability, no clinical responses	NCT00445913	(44)
	Citrullinated peptide loaded DC, intradermal	RA	Safety and tolerability, no clinical responses	N.A.	(45)
	VitD3/dexa synovial fluid loaded DC, intra-articular	RA	Safety and tolerability, Knee symptoms stabilized in two patients	NCT01352858	(46)
	VitD3 myelin peptides loaded DC, intradermal	MS	Ongoing	NCT02618902	N.A.
	VitD3 myelin peptides loaded DC, intranodal	MS	Ongoing	NCT02903537	(47, 48)
	Peptides loaded TolDC i.v.	MS neuromyelitis optica	Ongoing	NCT02283671	(47–49)
	IFN-α/GM-CS/Dexa DC, intra-articular	RA	Ongoing	NCT03337165	N.A.

*MBP, Myelin Basic Protein; PPI, PrePro Insulin; hINS, Insulin; Ins, Insulin; GAD, glutamic acid decarboxylase; APL, Altered Peptide Ligand; PLGA, poly(lactic-co-glycolic acid; VitD3, Vitamin Da; Dexa, dexamethasone; MS, Multiple Sclerosis; T1D, type 1 diabetes; FDR, first-degree-relative; LADA, latent autoimmune diabetes; RR-MS, relapse and remitting MS; CD, Celiac Disease; CD-GFD, Celiac Disease in gluten-free-diet; SLE, Systemic Lupus Erythematosus; IBD, Inflammatory Bowel Disease; RA, Rheumatoid Arthritis; i.m., intramuscular injection; s.c., subcutaneous; i.v., intravenous injection; i.p.,intraperitoneal injection; N.A., not applicable*.

## Cell Free Strategies

Inverse gene vaccination strategies aim at the induction of tolerance to a relevant Ag by means of transient expression of whole proteins or epitopes from DNA or RNA vectors in the absence of pro-inflammatory stimuli. Once injected, the coding sequence needs to enter the cytoplasm of the target cells, and, in case of DNA vectors, translocate to the nucleus for transcription, followed by translation in the cytoplasm and presentation of the Ag in the context of HLA class I molecules. The balance between an inflammatory immune response and the induction of tolerance can be controlled by several factors, including the route of administration, the target tissue, and the vector design. For example, direct transfection or transduction of professional antigen presenting cells (APCs) may result in efficient presentation to Ag-specific CD8^+^ T cells (50) or, as a consequence of cell death or tissue damage, the Ag may be taken up by professional APCs, processed as exogenous Ags, and presented to Ag-specific CD4^+^ T cells in the context of HLA class II (51, 52). On the same line, the vector backbone itself may contain immunostimulatory sequences, which could impact on gene expression, intracellular localization of the product and APCs activation *via* TLRs (53). Hence, the activation status of APCs is pivotal for the final outcome of the response: protection vs. tolerance. Two strategies for the delivery of the Ag-coding sequences have been used in preclinical studies, plasmids and viral vectors [reviewed in (42)].

### Plasmid DNA

Intramuscular plasmid DNA vaccination has been the most studied, likely due to the short persistence in the host, the low immunogenicity, and the low costs of plasmid production. This strategy was first tested in experimental autoimmune encephalitis (EAE), the murine model of multiple sclerosis (MS): immunization with plasmid encoding for an EAE epitope of myelin basic protein (MPB) prevented disease development, via T helper (Th)2 cell skewing of the Ag-specific T cell response (54). The initial preclinical studies led to clinical testing of this strategy not only in MS (4, 5), but also in Type 1 Diabetes (T1D) (6) (Table 1). A DNA vaccine (BHT-3009, Bayhill Therapeutics) containing full-length sequence of the human MBP was tested in two trials in MS patients (4, 5). In the first trial no severe adverse events were reported. Results indicated a trend of lower lesion activity, reduced IFNγ-producing CD4^+^ T cells up to 50 weeks after initiation, and a decrease of autoantibodies in the cerebrospinal fluid (4). Nonetheless, in the second trial the intervention did not result in any differences in the time to first relapse, rate of relapses per year, disability progression, and the treatment showed a deleterious effect at high vaccine dose, likely due to a greater percentage of immunostimulatory CpG motifs in the DNA plasmid (5). A similar approach was tested in T1D with a bacterial plasmid encoding for pro-insulin [BHT-3021, Bayhill Therapeutics; (6)]. No serious adverse events were observed, and the treatment resulted in improvement of endogenous insulin production, measured as 28% increase in C-peptide, and decreased frequency of proinsulin-reactive CD8^+^ T cells (6). Despite encouraging results, insulin requirements did not change substantially, and demonstration of efficacy is still pending. The same product (under the name TOL-3021, Tolerion Inc.) is going to be tested in two distinct phase II trials in T1D children and adults (NCT03794960 and NCT03794973). On the same line, DNA vaccines based on oral administration of recombinant live attenuated bacteria expressing diabetes autoAgs in combination with inhibitory cytokines, such as transforming growth factor (TGF-β1) and IL-10 or with anti-CD3 mAb have also been tested to prevent or revert the onset of diabetes in non obese diabetic (NOD) mice, showing induction of Tregs (both FOXP3-expressing and Tr1 cells) and suppression of autoimmunity (55, 56). A phase I trial will test the safety of subcutaneous injection of a plasmid co-encoding for T1D Ag and adjuvant cytokines (NNC0361-0041: plasmid encoding pre-proinsulin, TGF-β1, IL-10, and IL-2, Novo Nordisk A/S, NCT04279613).

Overall, thus far the plasmid DNA delivery approach showed the ability to skew the immune response, with no evidence of stable tolerance induction. The combination with immunomodulatory cytokines, which should sustain Ag-specific Treg induction, is expected to boost the induction of active tolerance. Results of ongoing clinical trials will shed light on the valuability of this approach.

### Viral Vectors

As alternative to plasmids, the use of viral vectors allows to restrict expression of the autoAg to specific tissues and avoid unwanted expression in activated APCs. In this context, the liver is an ideal target, due to its intrinsic tolerogenic properties [reviewed in (57)]. Two types of viral vectors have been used to target gene expression specifically to hepatocytes: the recombinant adeno associated vectors (AAV) and the lentiviral vectors (LVs). Although widely used as vector systems for liver directed *in vivo* gene therapy, few groups explored the use of AAV to induce tolerance to autoAgs in autoimmune diseases. Liver gene therapy with an AAV vector encoding for the full sequence of myelin oligodendrocyte glycoprotein (MOG) prevented development of and reversed preexisting EAE *via* the induction/expansion of Ag-specific FoxP3^+^ Tregs (58). Earlier studies of intramuscular injection in NOD mice of AAV encoding for glutamic acid decarboxylase (GAD) peptides prevented the development of overt diabetes in NOD mice *via* skewing of Teff cells to Th2 responses, but those studies were not further developed and active tolerance was not demonstrated (59).

The use of LVs to induce Ag-specific tolerance upon liver targeting was also investigated in NOD mice. Intraveneous injection of LV encoding the insulin B chain (InsB) 9–23 epitope led to specific expression of the autoAg in hepatocytes, thanks to the use of tissue-specific promoter and concomitant de-targeting of Ag expression in professional APCs by miR142 target sequences. This treatment prevented diabetes development by induction of Ag-specific FoxP3^+^ Tregs. Although highly efficient in prevention, the control of overt disease required a combination therapy with anti-CD3 mAb, to block Teff cells from destroying the target organ (60).

Gene vaccination strategies present several advantages in terms of cost-efficient production and long shelf life for plasmid-based vaccines and available (although expensive) large scale and clinical grade protocols for LV production. However, administration of the therapeutic products invariably leads to deleterious activation of professional APCs and the innate immune system (61) and may not be sufficient to counteract the burden of expanded Teff cells with multiple Ag-specificity. The future of these approaches points at combined therapies to overcome these hurdles.

### Protein Delivery Approaches

The direct administration of autoAgs in non-inflammatory conditions to induce tolerance in T cell mediated diseases has been widely investigated, especially in EAE and NOD pre-clinical models (62, 63). The underlying idea is that repetitive administration or exposure to large amounts of protein Ag, as whole protein, native or altered peptide alone or combined to carrier complexes, in the absence of pro-inflammatory adjuvants, will favor the deletion or clonal anergy of autoreactive Teff cells and the induction of Ag-specific Tregs, *via* uptake and presentation of the Ag by endogenous tolerogenic APCs (62, 63). In this context the route of administration is a key issue: the positive results obtained in allergic diseases by oral, intranasal and subcutaneous administration of allergens [reviewed in (64)] led to parallel attempts in autoimmune diseases.

Due to the early recognition of insulin epitopes as antigenic targets in NOD mice (65), insulin was the first Ag investigated for the development of protein-based immunotherapy of T1D. Initial promising results in murine models (66–68) led to the clinical testing of oral (7–10) and intranasal insulin [(11, 12), and INITII, NCT00336674], as tolerizing protocols in subjects at risk to develop the disease [(10, 13), NCT00336674 and TN20, NCT02580877] or in recent onset T1D patients (7–9, 11) (Table 1). Although results of few trials are still unpublished (NCT00336674; NCT02580877), thus far, none of them resulted in preserved insulin secretion in T1D patients. Inverse vaccination with InsB has also been tested as intramuscolar injection with Incomplete Freund's Adjuvant [IBS-VS01, (14)]: despite induction of InsB-specific Tregs, C-peptide levels were unaltered by the treatment. A new formulation of the vaccine in combination with MAS-1, an emulsion-based adjuvant, known to promote Th2 responses (69), is currently being tested in a Phase I study (MER3101, NCT03624062). Several trials betted on GAD65 as key Ag and on different routes: Dyamid, a GAD-Alum vaccine, was administered subcutaneous in recent onset T1D (15, 16) and in adults with latent autoimmune diabetes (LADA) (17) without achievement of clinically desirable results (18). Combination of Dyamid with vitamin D in LADA is currently being tested in a Phase II trial (NCT04262479). Similarly, attempts of oral tolerization with myelin Ags in MS, which date back to the early 90's, showed modulation of Ag-specific immune response, but no evidence of efficacy (19, 20).

### Peptide Delivery Approaches

In parallel to whole protein-based approaches, administration of peptides derived from disease-causing Ags was also tested both in T1D and MS (Table 1). Intradermal administration of a HLA-DR4-restricted native peptide derived from proinsulin (C19-A3) allowed maintenance of C-peptide levels in new-onset T1D over a 6-month treatment and resulted in increased frequencies of IL-10-expressing T cells [MonoPepT1De, (21, 22)]. The HLA-DR2-restricted immunodominant synthetic peptide MBP8298, containing the MBP immune-dominant epitope 85–96, was extensively tested in patients with MS, without stable clinical benefit (23, 24).

Overall these initial peptide-based approaches resulted in modulation of Ag-specific immune responses, but poor clinically relevant results, likely because autoimmune diseases are not caused by single T cell clones, as a result of epitope spreading (70). Given this phenomenon, recent studies have pointed at mixture of peptides from multiple autoAgs for the modulation of autoimmune diseases: in the context of T1D the MultiPepT1De (NCT02620332) and the IMCY-0098 trial (NCT03272269) have been completed, although results are still unpublished. The same approach was tested in MS: following promising results in humanized mice (25), the MBP-derived peptide cocktail ATX-MS-1467 (Aptiope (https://apitope.com/multiple-sclerosis/) was tested for safety and efficacy in relapsing MS patients. Results showed association of treatment with reduction in Magnetic Resonance Imaging (MRI) lesions (26). Moreover, transdermal application of a mixture of 3 myelin peptides showed significant effect in reducing the MRI and clinical outcomes (27) via the induction of Tregs (28). Similarly, NexVax2, composed of three HLA-DQ2.5-restricted immunodominant gliadin peptides, NPL001, NPL002, and NPL003, has been tested in a phase I clinical trial in Celiac Disease (CD) patients. Despite some gluten-related gastrointestinal side effects, the treatment was safe and well tolerated (29, 30). In treated patients functional unresponsiveness of T cells after gluten challenge was observed, indicating induction of tolerance. Currently, a phase II quadruple blind clinical trial (NCT03644069) is underway (71).

Modification of native peptides alters the way peptides interact with TCR and, therefore, influences subsequent T cell activation and T cell fate. Increasing knowledge of both MHC binding registers and TCR interacting residues of peptides allowed the development of altered peptide ligands (APL), with the aim of favoring the expansion and/or induction of Tregs upon peptide recognition [reviewed in (72, 73)]. Following studies in murine disease models, showing that specific APLs were capable of eliciting cytokine release and affecting T cell polarization (74, 75), APLs were tested *in vivo* in autoimmune diseases. Indeed, two altered peptides of MBP83-99 have already been tested in MS. NBI-5788 (Neurocrine Biosciences Inc), in which L-amino acids were changed to D-amino acids at positions 83, 84, 89, 91, was known to stimulate Th2-type responses in MS patients' PBMC (76). Clinical testing confirmed Th2 immune deviation in treated patients (31), but several patients developed hypersensitivity and antibodies that cross-reacted with native MBP83–99 peptide (32). The second MBP-derived APL tested is CGP77116 (Ala D-amino acids at positions 83, 84, 89, 91). It caused a Th1 skewing of CD4^+^ T cells cross-reacting with the native peptides, thus raising issues on the APL design (33). On the same line, the use of an insulin β-chain-derived APL (NBI-6024) did not improve or maintain beta cell function in recent onset T1D patients (34). Despite the promising results obtained in murine models and the improvements in the development of algorithms for peptide-HLA-binding prediction, thus far clinical trials using APLs were unsuccessful. The design of APLs currently represents a major caveat: the ability to predict the consequence of peptide binding to HLA molecules on APCs or Ag-receptors on T and B cells is still limited and it needs to be empirically determined for each peptide.

Regardless of the type, origin, number of Ags or the route of administration used, the outcome of the administration of whole proteins or peptides is strictly dependent on the activation status of the host's APCs the Ag is binding to. APCs *in vivo* exist in several different flavors (77), expressing different ranges of activatory or inhibitory cell surface molecules and soluble mediators, which play a critical role on the outcome of the cognate T-APC interaction. Peptide-based therapy showed immunological effects, including increased frequency of Treg cells and of IL-10, suggesting modulation of pathogenic responses. The beneficial effects, although observed only short-term after treatment, are compatible with immune tolerance, thus suggesting that endogenous APCs function was modulated, likely indirectly by a bystander suppression mechanism. We believe that to better sustain long term tolerance protein or peptide based approaches could benefit from strategies designed to keep APCs in check.

One of the strategies tested to address this limitation is the injection of soluble peptide-MHC (pMHC) complexes to target directly T cells. Vaccination with pMHC complexes is predicted to induce tolerance either by deletion of naive and memory Teff cells that recognize the self-peptide, or by induction of Tregs (78). This strategy was applied in preclinical models of Myastenia Gravis (79), in EAE (80–82) and in NOD mice (83) resulting in reduction of T cell responsiveness. Phase I trials were performed in MS patients (35, 36), but further testing is necessary to assess clinical efficacy.

The experience with administration of soluble Ags was further developed using different types of vehicles designed to deliver the Ag specifically to steady-state or tolerogenic APCs, as outlined below.

## Vehicle Approaches to Deliver Antigens

A number of different approaches to deliver Ag specifically to APCs *in vivo* have been investigated in pre-clinical studies and some of them have been translated into clinical application.

### Peptide Coupled to Cells

The first approach tested was the intravenous administration of antigenic peptides cross-linked to peripheral blood or splenic leukocytes using 1-ethyl-3-(3-dimethylaminopropyl) carbodiimide (ECDI), which promotes Ag coupling and induces cell apoptosis (Ag-SP) (84). Once injected *in vivo*, apoptotic Ag-SP are taken up by APCs and trigger the production and secretion of IL-10 and TGF-β and the up-regulation of PD-L1, leading to T cell anergy and apoptosis of pathogenic T cells and Treg induction (84). The efficacy of Ag-SP has been demonstrated in pre-clinical models, including EAE and NOD mice [reviewed in (84)]. The translation to the clinic of this approach was the administration of autologous peripheral blood cells coupled with seven MS-related peptides to MS patients in a Phase I-II clinical trial (ETIMS) (Table 1). Results demonstrated the feasibility, safety and tolerability of the treatment, and a decrease in Ag-specific T cell responses (37).

An alternative approach to deliver Ags to APCs in a tolerogenic manner is the administration of Ag-loaded erythrocytes, thus exploiting the natural tolerization mechanisms of dying red blood cells (85). To facilitate the binding of peptide Ags to erythrocytes, peptides were designed to contain the 12aa sequence ERY1 that binds to glycophorin A or sortase A on erythrocytes. Testing in EAE, in NOD and in transgenic mice demonstrated the deletion of Ag-specific T cells *in vivo* (86–88). Using this approach a phase Ib trial (ETIMS^Red^) has been completed; mechanistic studies demonstrated a reduction in myelin-specific T cell responses with an increased frequency of Tr1 and nTreg cells, thus pointing toward active induction of immune tolerance (38). Possible clinical translation of the erythrocyte binding technology is currently pursued by Anokion (www.anokion.com).

### Liposomes and Nanoparticle

As an alternative, to mimic the features of apoptotic cells, liposomes containing phosphatidylserine have been developed and loaded with antigenic peptides. Injection of liposomes loaded with MS-related peptides reduced symptoms in the EAE model (89). Instead, phosphatidycholine liposomes loaded with Ag and NF-kB inhibitors reduced disease severity in a mouse model of arthritis (90). Poly-(lactic-co-glycolic acid) (PLGA) microspheres carrying anti-sense oligonucleotides for the costimulatory molecules CD40, CD80, and CD86 delivered to NOD mice prevented T1D development (91, 92). Notably, the Authors showed that the Ag was not required to elicit Ag-specific Tregs, since, upon microsphere administration, DC migrate from the site of injection to the pancreatic lymph nodes, where auto-Ags are captured and presented to T cells, thus leading to Ag-specific Treg induction (91). This approach is under development for the treatment of T1D (DiaVac. Inc, https://www.angelmd.co/en/startups/diavacsinc).

The discovery that polymeric biodegradable nanoparticles (NPs) could efficiently deliver molecules *in vivo*, prompted investigators to develop NPs suitable for tolerance induction. PLGA-NPs can encapsulate immune-modulatory agents, such as rapamycin, alone or in combination with peptide Ags. Once injected *in vivo* these NPs target DC, thus allowing Ag-presentation in a tolerogenic manner (93). Pre-clinical studies showed that *in vivo* delivery of PLGA-NPs containing MS-related peptide Ags prevents and treats EAE by up-regulating PD-L1 on APCs and inhibiting the production of pro-inflammatory cytokines by Ag-specific pathogenic T cells (93). PLGA particles encapsulating gliadin (TIMP-GLIA) were developed for application as a therapy for CD and tested in a Phase I clinical trial (NCT03486990). The results of this trial are yet to be published, and the Phase II trial is currently underway (NCT03738475).

The tolerogenic effects of NPs depend on size, which dictates their trafficking and biodistribution: (i) particles smaller than 6 nm drain to the blood; (ii) particles larger than 9 nm preferentially drain to lymphatics; (iii) particles in the range of 20–100 nm accumulate in liver sinusoidal endothelial cells (LSECs) or macrophages; (iv) particles from 100 to 200 nm can traffic to the spleen and liver; (v) particles from 200 nm to 5 μm accumulate in the spleen. Moreover, NPs biodistribution is also affected by the route of administration: intravenous injection targets APCs in the spleen and liver, whereas upon subcutaneous injection NPs are taken up by DC that accumulate in draining lymph nodes (94). The ability of LSECs to promote induction of FoxP3^+^ Tregs, prompted the development of NPs to deliver Ags to LSECs for autoimmune disease treatment (95). NP-based autoAg delivery to LSECs prevented the onset of clinical EAE and, in therapeutic settings, mice with already established EAE improved rapidly (95).

More recently, a further evolution on the NP approach to deliver Ag and promote tolerance was described by Santamaria et al. (96, 97). This approach consists on coating NPs with MHC class I or MHC class II molecules coupled with antigenic peptides (pMHC-coated NPs) (98). In pre-clinical models, the administration of pMHC-coated NPs promoted the differentiation of Ag-specific Tr1 cells and the conversion of Ag-specific Th1 cells into Tr1 cells, following massive expansion. Expanded Tr1 cells were activated by autologous APCs presenting the cognate Ag and induced bystander IL-10-mediated suppression (98). These pMHC-coated NPs (Navacims™) have been validated in different pre-clinical models of autoimmunity (96, 97) and are currently under clinical development.

The application of nanotechnology to advance treatment of autoimmunity is likely to undergo major development in coming years. Nanotechnology will create new materials for NP-related products. However, NPs are highly reactive, leading to their potentially harmful interaction with biological systems and the environment, thereby increasing the risk of toxicity. Detection of adverse effects is complex, since they depend on the route of administration, doses and size of NPs. NPs accumulate in the reticuloendothelial system and their long-term effects are not yet fully elucidated. Moreover, the small size of nanomaterial allows their penetrance into deeper areas of biological systems that are usually inaccessible to larger particles. Thus, due to the different properties of NPs, their application for therapeutic purposes, especially the long-term effect on the immune system, requires further attention and research (99, 100).

## Cell-Based Approaches

Cell-based therapies are clinically attractive for promoting or restoring tolerance in T cell mediated diseases as they can theroretically control several inflammatory cells, including T and B lymphocytes, NK cells and APCs, leading to the control of unwanted immune responses. Therapies based on adoptive transfer of regulatory cells (T, macrophages, and DC) entered the clinical trial arena in the last years with the goal to investigate the safety and feasibility of the approach, and several studies are still ongoing.

### Treg-Based Therapies

The increasing knowledge on the biology of Tregs, on their mode of action and their ability to control autoimmune responses when adoptively transferred *in vivo* in pre-clinical models of autoimmunity allowed the growth of a number of clinical trials to investigate the safety and feasibility of the approach (42, 101). The literature on Treg cell therapy is extensive and will not be reviewed here in depth. Tregs were first used in clinical trials to treat patients with graft vs. host disease (GvHD) after hematopoietic stem cell transplantation (HSCT). Results demonstrated that Tregs are safe, with some concern about the occurrence of mild to moderate infections (101). Treg therapy is currently applied to reduce dependency on immunosuppressive drugs in patients after organ transplantation (101, 102). In the context of autoimmune diseases both FOXP3^+^ Tregs and Tr1 cells have been tested in clinical trials (Table 1). The infusion of *ex-vivo* expanded polyclonal FOXP3^+^ Tregs in patients with recently diagnosed T1D showed improved beta-cell function and reduced exogenous insulin requirement only short-term (39–41). The limited efficacy of Treg-based immunotherapy in T1D may depend on the limited number of residual functional beta-cells at time of treatment, the inadequate availability of IL-2 *in vivo* (40), or, more importantly, on the lack of antigen-specificity of the infused Tregs. A number of clinical trials with expanded autologous Tregs are ongoing, have been closed, or have been completed but results have not been published yet [NCT02428309; NCT02494492; NCT02691247; NCT02704338; NCT03239470; NCT03185000; NCT03773328; NCT03865017; (42)].

Pre-clinical studies indeed showed that Ag-specificity may offer an advantage for Treg function compared to polyclonal Tregs (103). The first experience with Ag-specific Tregs was in Crohn's disease: ovalbumin-specific Tr1 cells (Ovasave^®^) expanded *in vitro* were infused in patients, who ingested ovalbumin to allow Treg activation and inhibitory function in the gut, with no side effects, but limited clinical effects (43). Beside the use of T cell clones, several other approaches have been investigated and applied to generate Ag-specific Tregs. The most advanced strategies were applied to the transplantation area: alloAg-specific Tregs can be generated using tolerogenic DC (104–106) or by engineering Tregs with a chimeric antigen receptor (CAR) recognizing HLA-A2 (107, 108). These approaches are currently under clinical investigation (NCT03198234; TX200, www.sangamo.com). Translation of the latter strategy to autoimmune settings is more challenging because (i) the Ags inducing the disease are often unknown; (ii) Tregs and pathogenic T cells are driven by different epitopes; and (iii) while disease progresses, epitope spreading occurs.

Results of the pioneer trials of adoptive Treg cell therapies in transplantation and T1D taught the field that transfer of Tregs alone may not be sufficient to control immune responses in the long-term, thus combined therapies with growth factors or repetitive Treg injections are currently under investigation. Based on the evidence that low doses of IL-2 can increase the endogenous pool of Tregs (109), the combination of a single infusion of autologous *ex-vivo* expanded polyclonal Tregs with IL-2 or with Liraglutide in patients with T1D is currently under clinical testing (NCT02772679 and NCT03011021).

Overall, Treg-based clinical trials demonstrated the safety and feasibility of the approach with some clinical benefit. However, several open issues remain to be solved specifically in the application of polyclonal *ex-vivo* expanded Tregs: (i) their potential to mediate pan immunosuppression *in vivo*, due to the phenomenon of bystander immune suppression; (ii) their intrinsic instability when exposed to strong inflammatory conditions *in vivo*, thereby the risk of pathogenic conversion and exacerbation of the disease; (iii) the overall impact of long-lasting Tregs on infections and malignancies (110).

### DC-Based Approaches

It is now widely accepted that DC, either naturally arising or experimentally induced, play a critical role in the maintenance of tissue homeostasis and in promoting tolerance [reviewed in (111–113)], thus acting as regulatory cells. DC can acquire regulatory capacity upon treatment with immunosuppressive mediators, genetic manipulation or signals from other immune cells (114). DC with regulatory properties are generally indicated as tolerogenic DC (tolDC): they present Ags and prime Ag-specific T cells, while down-regulating the expression of costimulatory molecules and pro-inflammatory cytokines, and up-regulating the expression of inhibitory and/or modulatory receptors and anti-inflammatory cytokines. As a result, priming or activation of T cells by tolDC leads to induction of Ag-specific Tregs (114). On the other hand, DC sense environmental signals, which can impact their maturation and activation status and can modulate their microenvironment by release of soluble factors, thus indirectly impacting the outcome of Ag recognition by T cells.

**Figure 2:**
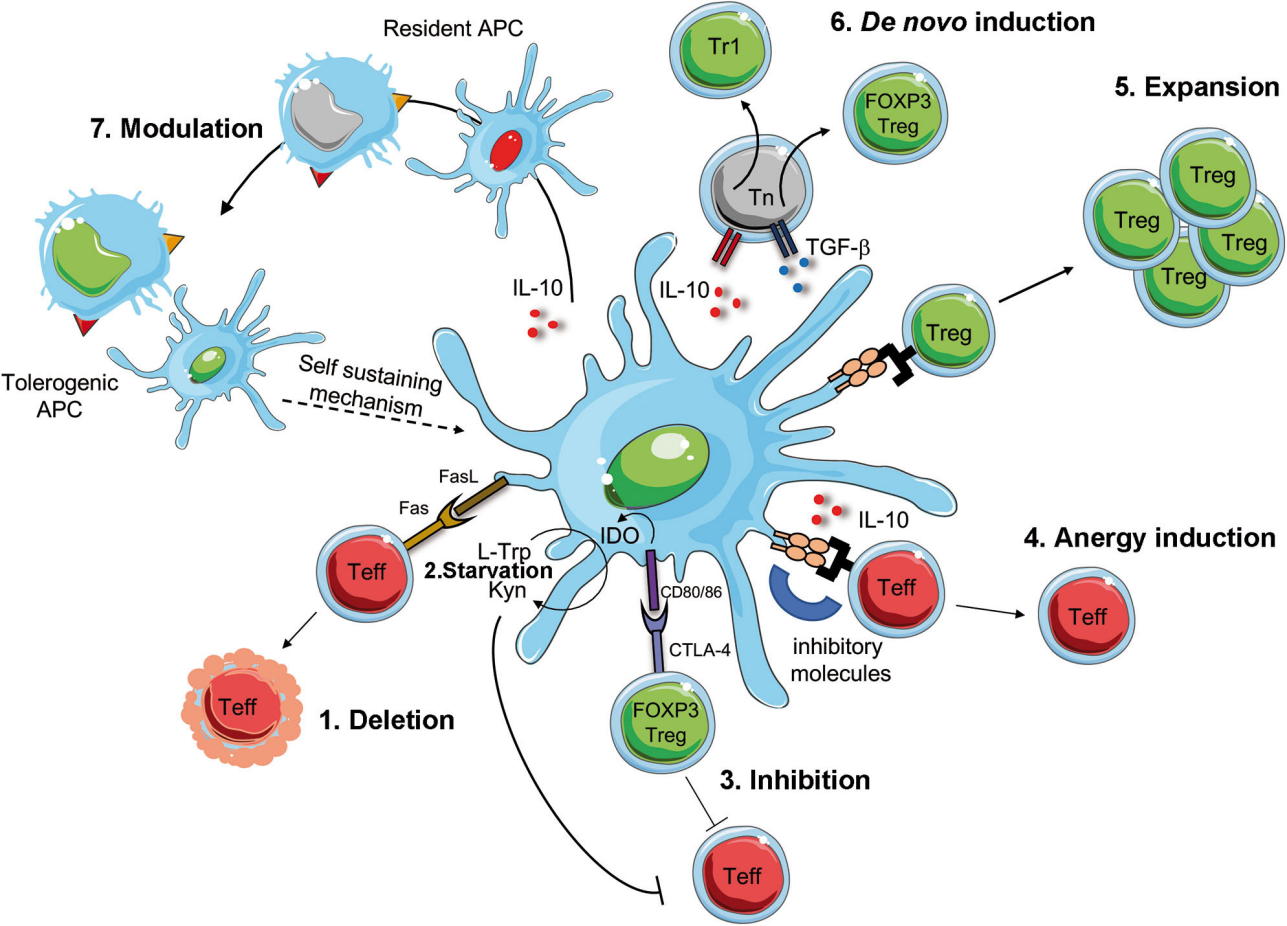
Mechanism of DC-mediated tolerance. Tolerogenic (Tol)DC promote deletion or modulate Teff cells via Fas/FasL interaction [1], starvation of Teff cells *via* IDO production that degrades tryptophan (L-Trp) into kynurenine (Kyn) [2]. IDO is induced by the interaction between CD80/CD86 on tolDC and CTLA-4 on regulatory T cell (FOXP3 Treg), which concur to the suppression of Teff cells [3]. The interaction of inhibitory molecules on tolDC and Teff cells in the presence of IL-10 secretion promotes T cell anergy [4]. TolDC favor the activation and expansion of pre-existing Tregs [5] of *de novo* induction of FOXP3 Treg of Tr1 cells [6]. Finally, surface expression of inhibitory molecules and secretion of regulatory mediators promote the conversion of resident APCs into tolerogenic APCs, which sustain tolerance [7]. Teff, effector T cells; CTLA-4, cytotoxic T-lymphocyte antigen-4; IDO, indoleamine 2,3-dioxygenase; L-Trp, L-tryptophan, Kyn, kynurenins; Fas, first apoptosis signal; FasL, Fas ligand.

A better understanding of the biology of tolDC and the development of protocols for the generation of tolDC *in vitro*, opened the possibility to translate their use as immunotherapy in clinical trials for immune-mediated diseases (115, 116). These therapies are not simple alternatives to Treg-based therapies, but they are complementary. *Ex-vivo* generated tolDC have the potential to induce, enhance, or restore Ag-specific tolerance *in vivo* since, once loaded with Ags, they act in an Ag-specific manner. TolDC can regulate pathogenic T cell responses *via* several mechanisms, including T cell deletion or inhibition, induction of T cell anergy, *de novo* Treg generation or expansion of pre-existing Tregs, and modulation of APCs (Figure 2). TolDC can delete Teff cells by inducing T cell apoptosis *via* Fas/FasL pathway. Furthermore, tolDC can inhibit Teff cell function either directly, *via* production of the enzyme indoleamine 2,3-dioxygenase (IDO), which degrades the amino acid tryptophan (L-Trp) causing starvation of pathogenic T cells (117), or indirectly, by activating pre-existing Tregs *via* interaction between CD80/CD86 and CTLA-4 to exert their suppressive function. TolDC can also promote the induction of T cell anergy into Teff cells *via* the secretion of anti-inflammatory cytokines, such as IL-10, or signals *via* inhibitory molecules, such as HLA-G and ILT3/4 (104, 118). Moreover, tolDC promote the expansion of pre-existing Tregs and *de novo* induction of both Tr1 cells and FOXP3^+^ Tregs, *via* the secretion of IL-10, TGF-β and active kynurenines, products of IDO-mediated L-Trp degradation (119). Finally, tolDC, via the expression and secretion of regulatory molecules, can also modulate APCs, rendering them pro-tolerogenic (e.g., modulation of resident macrophages into an M2 phenotype, or dampening the maturation of resident DC), a process that generates a self-sustaining tolerogenic microenvironment, which can promote long-term tolerance. Beside exerting their effect on immune cells, tolDC secrete several factors (e.g. pro-angiogenic cytokines) which promote tissue repairing and regeneration (Figure 2). Altogether, these properties rendered tolDC the cells of choice to restore tolerance in autoimmune diseases.

Pioneer clinical trials with adoptive transfer of tolDC demonstrated the safety, feasibility and efficacy of the treatment and some clinical benefits [reviewed in (115)] (Table 1). Several tolerogenic approaches have been used in the past. In the first-in-man study, autologous tolDC treated with antisense oligonucleotides targeting CD40, CD80 and CD86 to maintain their immature state were infused in T1D patients [NCT00445913, (44)]. The group of Thomas treated DC with a nuclear factor-kB (NF-kB) inhibitor and pulsed them with citrullinated peptide Ags before injection into RA patients (45). More recently DC differentiated with vitamin D3 and dexamethasone alone or in combination have been or are currently used to treat RA, Crohn's disease, and MS patients [NCT01352858-AutoDECRA (46); NCT02618902-MS-tolDC; NCT02283671-TolDecEM/NMO (47–49); NCT02903537-Tolervit-MS (48, 49); NCT03337165-TolDCfoRA; (120)].

Despite these encouraging results, phase II/III clinical trials are needed to address several open issues and to allow comparison to current available treatments. Indeed, a number of open questions remain before tolDC-based therapies can be routinely used to treat or cure autoimmune diseases (101, 116). A variety of routes for tolDC administration have been tested in the past, including intradermal, intraperitoneal, intravenous and intra-articular (121). These administration routes are indeed required to allow tolDC to reach the relevant draining lymph nodes or the disease-specific site of inflammation. However, if direct administration to the relevant tissue is challenging, such as in the case of T1D, intraperitoneal administration has been preferred.

As for any Ag-specific approach for tolerance induction, an additional major hurdle in developing an effective tolDC-based therapy is the selection of the Ag critical for a given disease. As in the case of peptide-based approaches, the use of broad spectrum disease-related peptides has been postulated to overcome this limitation [reviewed in (101)]. Interestingly, in the context of T1D the identification of neoepitopes opened new perspectives in the field. The peptides characterized by improved MHC binding register, such as the insulin peptide InsB_9−23_ with combined substitutions in positions 14, 21, and 22 (122), those generated by fusion of peptides, such as the Hybrid Insulin Peptides (HIPs) (123), or by aberrant translation, such as INS-DriP peptide (124), have been shown to trigger strong specific T cell responses. These highly immunogenic peptides presented by tolDC are promising tools for the reprogramming of pathogenic T cells and induction of tolerance in T1D.

Besides the critical issues discussed above some additional considerations should be taken into account when designing tolDC-based therapies: (i) the necessity of multiple cell infusions to allow the induction of the self-sustained mechanisms described above will invariably lead to high manufacturing costs; (ii) the generation of autologous tolDC implies the use of patient-derived monocytes, which may not be as functional as those isolated from healthy subjects (105); (iii) the stability of the cell product to be infused must be evaluated for limiting *in vivo* side effects or disease exacerbation.

## Conclusive Remarks

In recent years the development of *in vivo* and *ex-vivo* Ag-specific approaches to modulate detrimental immune responses has made striking progress. Results obtained in Phase I/II demonstrated the safety and tolerability of the approaches with, thus far, limited clinical responses. Phase II/III clinical trials will help in defining whether the strategies outlined here will reach the goal of completely reversing the course of T cell mediated diseases.

Overall, results obtained thus far highlighted common requirements for achieving the desired effectiveness of the Ag-specific based therapy, either peptide or protein delivery, or the vehicle strategies to delivery Ags or the regulatory cell-based approaches: the repetitive administrations and the use of multiple Ags to effectively activate the tolerogenic branch of the immune response and to tackle the epitope spreading, respectively. Moreover, the selection of the most suitable epitope/s to be used might be challenging, because different patients may display preferential response to specific Ags. This issue opens the need for the identification of peptide Ags that can be used across different HLA-type patients [e.g., (21, 22)] or for deeper characterization of patients' reactivity before enrollment in trials.

The field is rapidly evolving, and the upcoming clinical trials will confirm the safety and feasibility and will shed light on the efficacy of Ag-specific approaches. Several issues remain to be clarified for each of the approaches in the pipeline. Regardless of the tolerogenic approach used, one of the open questions in the field of tolerance induction is the definition of common parameters to monitor the response to treatment, and to allow comparison of different approaches. In the context of cell-based tolerance-inducing therapies an initiative of the European scientific community brought together the leader scientists in the field of cell-based therapies and autoimmune diseases under the umbrella of the European Cooperation in Science and Technology (COST). The main objective of A-FACTT Action was to coordinate efforts to minimize overlap and maximize comparison of the diverse cell-based approaches through establishment of consensus monitoring parameters (https://www.cost.eu/actions/BM1305/#tabs|Name:overview). More of such initiatives could help the field to address this relevant point. On the same line, the definition of “tolerogenic treatment” should be unambiguouosly referred to therapies inducing long-term active tolerance. Indeed, several treatments have been shown to modulate immune responses in the short term, but fail in controlling disease signs long-term. Tolerogenic therapies should promote long-lasting effects, and this can be achieved by different mode of action, including the conversion of pathogenic Teff cells into Tregs, or the *de novo* induction of Tregs. As discussed above, we believe that modulated DC, or APCs, represent the population of cells able to prevent activation of pathogenic Teff cells, to promote *de novo* induction of Tregs, and to re-educate Teff cells to become Tregs, thus maintaining tolerance long-term. To achieve these long lasting effects possible repetitive injection of the tolerogenic treatment might be required. Based on the central role of APCs in determining the outcome of Ag-specific T cell activation, inverse vaccination strategies are unlikely to be successful, unless the underlying mechanism allows boosting of the immunomodulatory properties of DC or, more generally, of APCs.

## Author Contributions

LP and SG wrote the manuscritpt. All authors contributed to the article and approved the submitted version.

## Conflict of Interest

The authors declare that the research was conducted in the absence of any commercial or financial relationships that could be construed as a potential conflict of interest.
